# Development of a Software and Hardware Complex for Monitoring Processes in Production Systems

**DOI:** 10.3390/s25051527

**Published:** 2025-02-28

**Authors:** Vadim Pechenin, Rustam Paringer, Nikolay Ruzanov, Aleksandr Khaimovich

**Affiliations:** 1 Research Laboratory ‘Artificial Intelligence in Production Systems’, Samara National Research University, Moskovskoye shosse 34, 443086 Samara, Russia; ruzanov.nv@ssau.ru; 2 Department of Technical Cybernetics, Samara National Research University, Moskovskoye shosse 34, 443086 Samara, Russia; rusparinger@ssau.ru; 3 Department of Engine Production Technology, Samara National Research University, Moskovskoye shosse 34, 443086 Samara, Russia; berill_samara@bk.ru

**Keywords:** camera, hardware–software complex, container, detection, neural network, algorithm

## Abstract

The article presents a detailed exposition of a hardware–software complex that has been developed for the purpose of enhancing the productivity of accounting for the state of the production process. This complex facilitates the automation of the identification of parts in production containers and the utilisation of supplementary markers. The complex comprises a mini computer (system unit in industrial version) with connected cameras (IP or WEB), a communication module with LED and signal lamps, and developed software. The cascade algorithm developed for the detection of labels and objects in containers employs trained convolutional neural networks (YOLO and VGG19), thereby enhancing the recognition accuracy while concurrently reducing the size of the training sample for neural networks. The efficacy of the developed system was assessed through laboratory experimentation, which yielded experimental results demonstrating 93% accuracy in detail detection using the developed algorithm, in comparison to the 72% accuracy achieved through the utilisation of the traditional approach employing a single neural network.

## 1. Introduction

Intelligent manufacturing is defined as the seamless integration of process automation and digital transformation. This integration involves the harmonious combination of industrial equipment with advanced sensor technologies [1], powerful computing platforms, and reliable communication systems. Such synergies have been shown to lead to significant improvements in production efficiency and overall management [2]. Flexible Manufacturing Systems (FMSs) can be defined as highly adaptable manufacturing systems capable of producing a wide range of products in varying volumes.

In the context of FMS operation, the challenges associated with managing incomplete production and the development of adaptive control methodologies within nonlinear systems become a salient issue [3]. Concurrently, the placement and movement of workpieces should be systematically documented during the FMS operation [4]. In scenarios involving the transfer of batches of workpieces between different facilities, such as between shops or between the FMS and CNC areas, the automation of tracking their composition and trajectory is attainable through the utilisation of machine vision technologies [5].

The primary function of MES systems is to monitor the execution of plans, thereby facilitating the collection and structuring of necessary information. Industries such as aerospace and shipbuilding are characterised by extended product cycles, necessitating the implementation of timely corrective actions in planning, which in turn requires enhanced objectivity in the control of material flows. However, it should be noted that MES systems are not capable of addressing errors related to the human factor. A significant challenge in contemporary manufacturing is the bridging of the data gap between planning and the actual production status.

Enterprises are increasingly turning to automated data collection and employing specialised software to monitor equipment operations. These systems [6,7] primarily collect data on equipment utilisation, while the registration of scrap and the technological composition of each individual part are conducted manually. Standards have been developed for the traceability of objects in production [8], with the objective of reducing errors in data collection and decreasing the time required for data collection by employing automation tools, particularly identification tags. In the context of parts moving in containers, it is not feasible to ensure the traceability of each part using identification tags.

The organisation of adequate tracking of material flows along the process chain allows for the detection of the location of each part in the batch. In this case, it becomes possible to assemble parts of different sizes united by one group of manufacturing technology into one batch. In order to realise this approach, it is necessary to follow the principle of unambiguous correspondence, ‘part ID–tare ID–cell ID in tare’. Given the potential variability of the tare along the technological route, necessitating its re-evaluation upon unloading, reloading, or placement of parts within or after the technological operation, the establishment of such a mutually unambiguous correspondence is imperative. Evidently, the efficacy of this detection process is contingent upon its automation with a high degree of reliability.

The identification of container IDs can be facilitated by the use of specialised tags, such as RFID [9] or QR. The former method necessitates the utilisation of dedicated readers, while the latter relies on visual accessibility and industrial cameras. In the context of RFID, it is imperative that each component is tagged, taking into account dimensional changes (i.e., material removal) that may occur during machining processes. The tag must be either radio frequency or attached to a separate ‘passport’ sheet. Conversely, QR employs a method that involves tagging the container directly or on a separate sheet. Machine learning algorithms [10,11], primarily convolutional neural networks [12], have been employed to identify the contents of the container. However, training neural networks capable of solving a wide range of problems, such as detecting multiple objects, necessitates substantial computational resources, extensive training data, and a considerable time investment. In light of these challenges, companies worldwide are expediting the development of innovative solutions, encompassing the creation of more efficient and specialised chips, as well as compact models that demand less power [13] or generate synthetic data.

## 2. Problem

**Figure 1:**
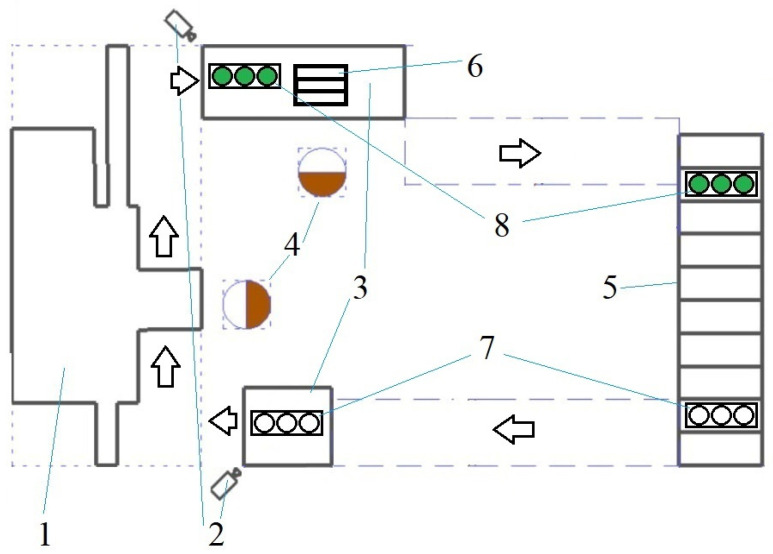
Example of a production cell. 1—processing equipment; 2—input and output cameras; 3—tables for placing containers with parts; 4—workers; 5—racks; 6—software and hardware complex; 7, 8—containers with workpieces before and after processing.

The following discussion will consider the video recording system, using the example of a ‘production cell’ consisting of one machine and racks, from where batches of parts come and where they return. The scheme of such a cell is shown in Figure 1.

The actions of the worker are described below, with particular reference to the data recording: (1) The container containing the parts is delivered; (2) The container is placed on the work table at the input, in front of the machine; (3) The container containing identical parts is placed on the table at the output of the processing equipment; (4) The parts are moved from the input container to the output container during the processing of parts; (5) In the event of defective parts, these are to be placed in the container designated for defective parts, which is located outside the camera area; (6) Upon completion of the batch of parts, the container at the input is to be removed, followed by the container at the output.

In the scenario delineated, upon the container’s sufficient displacement to deactivate the imaging system, a secondary camera is initiated at the exit connected to a second client application at the workplace. In the event of the presence of a container within the line of sight, the secondary camera will register events from it.

The process of locating the secondary camera (to which the server issues a signal to initiate activation) is facilitated by the location tree (the camera is assigned an identifier corresponding to the identifier of the location in the general tree, designated either as an entrance or an exit). When the container is moved out of the secondary camera’s field of view, the focus is redirected to the primary camera.

**Figure 2:**
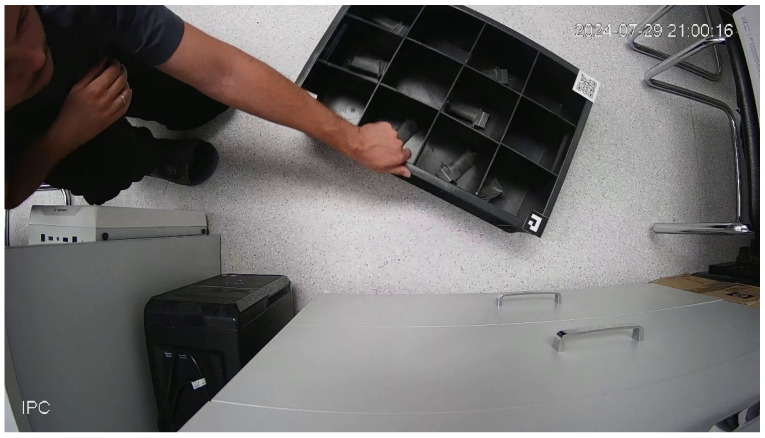
Example of ‘information noise’ on the frame.

The primary challenges that give rise to errors in this system pertain to random events that do not align with standard conditions, which can be designated as ’information noise’. Figure 2 presents a photograph captured from an IP camera, illustrating a series of such events.

The following components of ‘information noise’ have been identified: (1) The container is not located in a characteristic place (e.g., the floor as opposed to the table), thereby altering the background and distance to the object; (2) superfluous details are present in the frame (e.g., a person, a hand on the container, computers, chairs, a cabinet); (3) the container’s position is not aligned horizontally or vertically (e.g., at an angle); (4) the image of the container is partially out of the frame; (5) there is a violation of illumination.

The primary challenge in constructing an automatic video recording system for discrete production is the management of ‘information noise’. The necessity to train neural networks, which underpin the business logic of such systems, on limited samples represents an additional obstacle in successfully overcoming ‘information noise’. The objective of this article is to develop a hardware–software complex for video registration of discrete production in mechanical engineering, utilising machine learning technologies to diminish the human element involved in entering data on the state of production.

## 3. Materials

The experimental procedure involved the utilisation of two devices: a Logitech web camera (Logitech, Suzhou, China) and an IP camera model designated DH-IPC-HFW5442EP-ZE (Zhejiang Dahua Technology Co., Ltd., Hangzhou, China). These devices were employed for the purpose of documenting the experiments through photographic means.

The hardware components of the complex encompassed a system unit in the industrial version Hiper M9 Cel G5905 (3.5) 4 GB SSD128 GB UHDG 610, a Hama Action 165 3D tripod (Hama GmbH & Co., KG, Monheim, Germany) for the purpose of camera fixation, a communication module for the activation of automatic colouring and signalling to the operator, a signal lamp and an LED lamp for illumination, and I/O devices.

**Figure 3:**
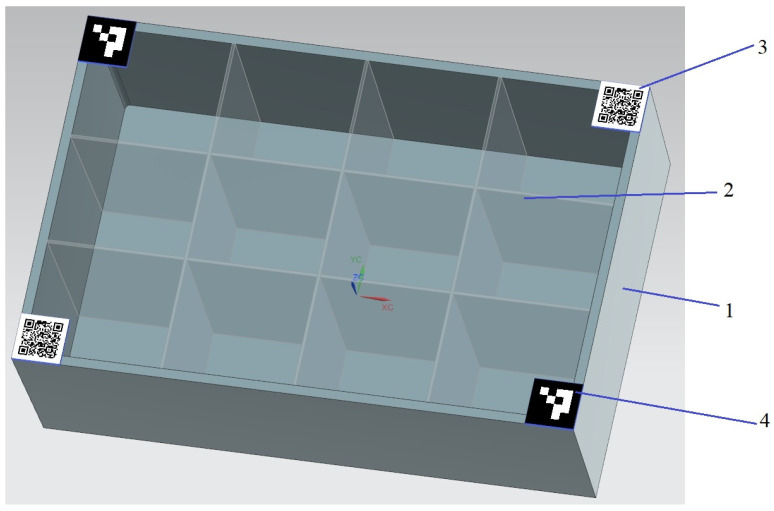
Design of the container for experiments. 1—case; 2—lattice; 3—QR code; 4—ArUco-marker.

Parts were moved in a container with a lattice; each cell could contain one part (or nothing). Figure 3 shows a 3D model illustrating the external characteristics of the aforementioned container.

As indicated by the markers situated at the corners of the container, automatic activation of the complex for content detection is facilitated by ArUco, whilst a QR code is employed for the analysis of information on the container and the selection of neural networks responsible for cell detection and the classification of parts of a certain type.

The training of neural networks was conducted on computers equipped with graphics processing units (GPUs).

To evaluate the accuracy of the detection results, the metric ${mAP}_{\left\{50-95\right\}}$ (Mean Average Precision) was used, which is the average of ten mAP metrics, which in turn is the average of all detected classes in the image for the AP (Average Precision) metrics for each class. AP is calculated for different IoU (Intersection over Union) thresholds from 0.5 to 0.95 in steps of 0.05. The equation for ${mAP}_{\left\{50-95\right\}}$ is as follows:

(1) $${mAP}_{\left\{50-95\right\}}=\frac{1}{10}\sum_{t=0.5}^{0.95}{mAP}_{t},$$

where $m{AP}_{t}$ is the value at an IoU threshold equal to t, and t takes values from 0.5 to 0.95 in steps of 0.05 (0.5, 0.55, 0.6, 0.65, 0.7, 0.75, 0.8, 0.85, 0.9, 0.95).

In the context of object detection, IoU (Intersection over Union) is a metric used to evaluate the accuracy of model predictions. It measures the intersection of two rectangles and divides it by the union of those rectangles.

The equation for calculating IoU is as follows:

(2) $$IoU=\frac{AoI}{AoU},$$

where AoI (Area of Intersection) is the area of the region where the rectangles intersect, and AoU (Area of Union) is the area of the figure that is the union of two rectangles.

If IoU is greater than the threshold, the predicted object is a true positive (TP). If IoU is less than the threshold, the predicted object is a false positive (FP). If an object present in the image is not detected by the network, it is classified as a false negative (FN). Any other region of the image (that does not contain an object and where the model did not predict it) can be considered a true negative (TN). Precision and Recall scores were calculated to assess the quality and also to calculate the average AP accuracy as follows:

(3) $$Precision=\frac{TP}{TP+FP},$$

(4) $$Recall=\frac{TP}{TP+FN},$$

Based on metrics (3) and (4), a *precision–recall* curve is constructed, and the area under its graph is calculated using the interpolated precision method [14]. The interpolated precision $p_{interp}(r)$ is calculated for eleven Recall values as follows:

(5) $$p_{interp}(r)=\underset{\tilde{r}:\tilde{r}\geq r}{\max}p\left(\tilde{r}\right),$$

where $r\in \left[0,0.1,0.2,\ldots ,1\right]$.

Finally, the average accuracy for each class is calculated as the arithmetic mean of the interpolated accuracies as follows:

(6) $$AP=\frac{1}{11}\sum_{\;r\in \left[0,0.1,0.2,\ldots ,1\right]}p_{interp}(r).$$

To calculate mAP for multiple classes, sum their APs and divide by the number of $N_{classes}$ as shown:

(7) $$mAP=\frac{1}{N_{classes}}\sum_{c\in classes}{AP}_{c}.$$

The accuracy of the classification process ac is determined by the ratio of correctly classified images, denoted by $N_{t}$, to the total number of images, denoted by $N_{all}$.

(8) $$ac=N_{t}/N_{all}.$$

The parameters of neural networks that have been the focus of this study were used to process the results of the experiments.

The following section will consider the hardware and software complex and the neural network algorithms and models that have been developed.

## 4. Methods

Currently, the most reliable tool for object detection in the presence of ’information noise’ is deep neural network models [15,16,17]. Such models can be used both for the detection of regions with markers and for the detection of the contents of production containers. Before conducting the experiments, we selected neural networks for detection.

### 4.1. Selection of Machine Learning Models for Object Detection

There are three main directions in the development of neural network-based object detectors: region-based networks (R-CNNs), single-channel moment detectors (SSD and YOLO), and transformer-based models (DETRs). These approaches offer different strategies for detecting and localising objects in images, each with its own strengths and applicability in specific environments. The choice of a particular method depends on speed, accuracy, and computational requirements, so an understanding of each of these areas is key to successfully solving computer vision problems.

#### 4.1.1. Region-Based Convolutional Neural Networks (R-CNNs)

An R-CNN [18] uses region proposals to search for potential objects in an image. The detection process consists of two steps: region of interest extraction and region classification by refining the coordinates of the enclosing rectangles. Fast R-CNNs [19] and Faster R-CNNs [20] improved the original architecture by integrating the region formation and classification process into one network, which speeds up the work and improves the accuracy.

The main advantage of an R-CNN is its high accuracy, especially for small and complex objects, and its flexibility, which allows the model to be adapted to different tasks (e.g., segmentation). However, these models are slow and require significant computational resources, which limits their use in real-time tasks.

#### 4.1.2. Single-Channel Moment Detectors

Within the class of single-channel moment detectors, there are two main models: the SSD [21] and the YOLO [22,23].

An SSD divides the image into a grid and predicts the coordinates of the bounding rectangles for each cell, using multiple levels of the feature pyramid to handle objects of different sizes. This provides high-speed performance, making the SSD suitable for real-time tasks, although it may have difficulty detecting small objects.

The YOLO also divides the image into a grid and predicts the coordinates of framing rectangles, but it is optimised for extremely high speed. This makes it ideal for use in mobile devices and tasks where speed is critical. However, a YOLO can lose accuracy in complex scenes and with small objects.

#### 4.1.3. Transformer-Based Detectors

A DETR [24] uses transformers to model the relationships between image regions, allowing for efficient object detection without complex post-processing. The model transforms an image into a set of features and applies attention mechanisms to predict the coordinates of framing rectangles and object classes.

A DETR is an innovative approach that integrates detection and classification in a single architecture. The main advantages of a DETR are high accuracy and the ability to handle complex scenes, but it requires significant computational resources and longer training times.

The YOLO family of architectures was chosen for the problem at hand, allowing for the results to be obtained faster and with a higher mean average precision (mAP) [25].

Three types of data are calculated as output from the YOLO network: classes of detected objects, coordinates of their bounding boxes, and their objectness scores. The loss function of the YOLO neural network used for detection thus has the following three components:

(9) $$L_{YOLO}=L_{cls}+L_{loc}+L_{conf},$$

where $L_{cls}$—classes loss; $L_{loc}$—location loss; $L_{conf}$—objectness loss.

The loss function uses binary cross-entropy to compute classes loss and objectness loss and the Intersection over Union metric to compute location loss. More detailed information about the $L_{YOLO}$ loss function in the YOLO can be found in [22].

### 4.2. The Developed Algorithm

Industrial enterprises with a wide range of production (300–400 types of parts in one production area) require an algorithm that is both fast (faster than a human) and reliable (less error-prone) for the identification of contents. The accuracy results of the YOLO network on such a limited sample may not fulfil these requirements, yet it significantly outperforms human performance. However, a YOLO trained to detect a specific class (e.g., QR tags) can achieve high accuracy in production environments and relatively small training samples. In view of the above, a cascade algorithm was developed, the main idea of which is to use one YOLO neural network to detect a specific class (ArUco, QR, cells of a specific container). Mathematical proofs regarding the necessary sample size for training a detection neural network when implementing such a cascade learning algorithm are given in Appendix A.

In order to enhance the precision of object categorisation within the cells, the implementation of an auxiliary neural network is recommended. This network is specifically designed for classification purposes, thereby minimising errors of the second kind (i.e., erroneous conclusions regarding the absence of objects) and enhancing the mean average precision (mAP) of the outcome.

With regard to the selection of the model, convolutional neural networks from the AlexNet [26], VGG family [27], MobileNetV2 [28], GoogLeNet [29], and ResNet [30] architectures were considered.

The VGG architecture was selected due to its simplicity and efficiency, and it has been demonstrated to be optimal in terms of the speed of operation and the accuracy of the results obtained [31].

The loss function for the neural network used in this paper for VGG19 classification is categorical cross-entropy.

(10) $$L_{CE}=-\sum_{i}^{C}m_{i}\cdot \log\left(s_{i}\right),$$

where C—number of classes; $m_{i}$—label value for the *i*-th case; $s_{i}$—value at the output of the neural network for the *i*-th case.

**Figure 4:**
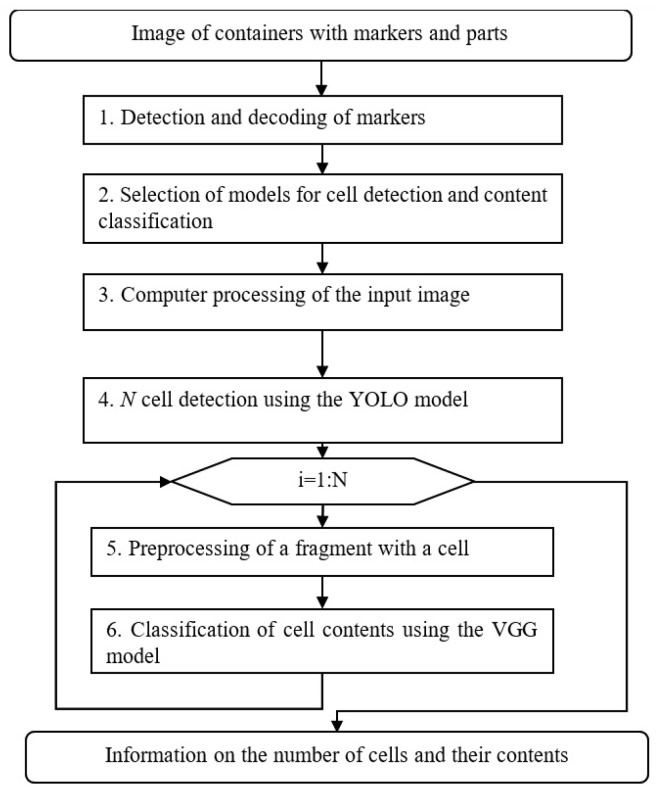
Cascade algorithm for container content identification.

In order to overcome the above-mentioned limitation of the volume of training samples, the study proposes the use of several neural networks trained to solve narrow tasks (the detection of a certain type of marker, cells, or classification within cells). Activation of the necessary neural network is possible if a special marker, QR, or other identifying code is attached to the object (a box with parts), which allows access to the system’s database, and on the basis of this information, the system activates the necessary neural network for further detection of objects. Figure 4 shows a block diagram of the developed algorithm stages.

The input image of containers with markers and parts is first passed through the marker detection algorithms: ArUco and then QR. A different YOLO neural network is used for each. In the next step, two neural network models are selected: YOLO, which is trained to detect the cells of a given container, and VGG, which is trained to classify the parts that are in a given container (based on the information in the database).

In the third step, computer processing (resizing) of the image is performed. In the fourth step, *N* container cells are detected using the YOLO neural network. All detected fragments containing cells are passed, in a loop, through the neural network for VGG classification. Before classification, the size and, if necessary, the colour mode are also resized. As a result of the first stage of the algorithm, information in dictionary format is produced: {‘result’: True/False, ‘items’: list of dictionaries for each label, ‘image’: image as an array of bytes with selected areas of labels}. The whole algorithm results in the formation of information in the form of a dictionary in the following format: {‘result’: True/False, ‘items’: list of dictionaries of the form {‘N’: cell number, ‘partClass’: part type, ‘type’: true/false}, ‘image’: image as a byte array with labelled cells and labels}. This generates the necessary data to populate up-to-date information about the status of the part batch.

### 4.3. Hardware–Software Complex Architecture

**Figure 5:**
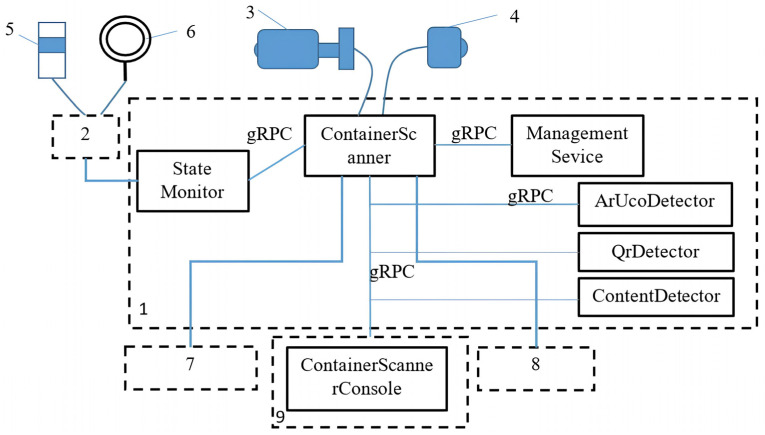
Hardware–software complex architecture. 1—Personal computer; 2—Communication module; 3—IP camera; 4—Web camera; 5—Signal lamp; 6—LED backlight lamp; 7—Input device; 8—Output device; 9—Portable computer (tablet).

The architecture of the developed hardware–software complex is shown in Figure 5.

All hardware components have been described in the Sensors section. As an input/output device, you can use a portable computer or mobile phone (9 in Figure 3), or you can use a keyboard, mouse, and monitor (7–8 in Figure 3) if you are working directly at the server computer 1. The communication module is a hardware device that connects to the computer via the USB port and is responsible for switching the LED and signal lights on and off.

The complex’s software consists of seven modules.

Management Service implements the business logic of the complex in terms of processing user requests and performing operations with the database. Management Service, in turn, can be divided into three main modules: the application server (Tomcat), the operations module, and the Data Access Object (DAO). Java Servlet technology was chosen for the implementation of the module.

Container Scanner is a module that manages data flows between hardware and computing services. It is implemented using the Java programming language (Java SE 17).

The cascade algorithm (Figure 4) is implemented using three microservices: ArUcoDetector (ArUco marker detection), QrDetector (QR marker detection), and ContentDetector (cell content detection). All microservices are implemented in the Python 3.11 programming language, and the OpenCV library and TensoFlow framework are used to implement image processing and neural networks. ArUcoDetector is responsible for sequentially reading data from the data source, detecting ArUco markers, and sending the data to the server for further processing using a cascade algorithm (Figure 4). The ArUco detector uses a buffer to store the last six consecutive frames. Since the event registration rate (number of frames per second) significantly exceeds the intervals of new events, such a system can be conditionally referred to the class of linear time-invariant (LTI) systems, which generate an output signal based on the input signal, taking into account the conditions of time invariance. In this case, the sliding window method we used provides the most adequate result of event interpretation, as it increases the algorithm’s robustness to information noise. In the framework of the algorithm, several frames in succession should give a stable (identical) result, and the change in the state of the system is carried out only in the case of a reliable result. While in the image processing loop, the ArUco detector can be in one of two states: EMPTY and DETECTED. The EMPTY state means that the detector has not detected any ArUco markers in two consecutive frames. The DETECTED state means the opposite. The transition between the states follows the following rules:

The detector starts in the EMPTY state.

It switches to the DETECTED state after ArUco markers have been detected on two consecutive frames. It then sends the last frame to the server for processing.

It remains in the DETECTED state until no markers are detected on two consecutive frames.

It sends the last frame from the buffer to the remote server for processing and enters the EMPTY state.

The QrDetector and ContentDetector process the data when ArUco detects markers on two consecutive frames.

State Monitor is a module that converts hardware–software complex state switching commands received via the gRPC protocol into control commands for signal and LED lamps that are understood by the communication module. The module has been implemented in Java.

The Container Scanner Console is designed to display a detailed status of the hardware–software complex, the current stage of information processing, and the manual correction of data on scanning results. The Java language was used during implementation.

The cross-platform *gRPC* framework is used to organise the interaction between modules. It enables data transfer between components using the *HTTP/2* protocol and the *Protocol Buffer* data format. The use of *gRPC* allows for a significant reduction in the delay and time of data transfer while maintaining a high degree of modularity and independence between different modules.

### 4.4. Single-Channel Moment Detector Training

The neural network detector YOLO was chosen as a base model for solving the problem of object detection, and then the task of training the neural network for automatic detection and recognition of several types of parts located in the cells of the container displayed on the test videos was solved. It is necessary to select and classify the cells of the container as empty or containing parts (with an additional definition of the part type), as well as to detect QR and ArUco markers located on the corners of the container (see Figure 3). A comparative study of different versions of this detector in the context of the task was carried out to select the best version.

The raw data comprised a series of videos displaying a person and two types of containers with cells. Each cell within these containers could contain one of several distinct parts, or alternatively, the cell may be empty.

Each video frame was then used to train the model. This process involved two key elements: the recognition of parts and the consideration of their type, as well as the type of QR marker (regular or ArUco).

#### 4.4.1. Data Cleansing

Firstly, data cleansing was performed to enhance the quality of the input data for training the neural network. To optimise the quality of the markup, video selection was conducted based on their quality metrics. The following steps constituted this process:

- Video cross-validation: A cross-validation method was used to assess the quality of the markup. Each video was successively taken as a validation (test) video, while the others were used to train the model. At the end of each training cycle, the detection quality of the validation video was evaluated. Videos showing low values of metrics (such as mAP, accuracy, and completeness) were excluded from further use.
- In addition, an attempt was made to enhance data quality by filtering individual frames based on the self-consistency criterion. If the detector predictions on a frame matched well with its partitioning, it was considered that the partitioning of that frame was of good quality. However, this approach did not result in a significant improvement, and it was decided to discontinue its use.

The initial sample consisted of ten videos. Following the cleansing of the data, six videos were selected for the training and test videos. The training sample contains 1906 frames, and the test sample contains 751 frames. The resolution of each frame was fixed at 640 × 480 pixels.

#### 4.4.2. Selecting and Training the Model

In the second step of the process, a comparative evaluation of different versions of the YOLO detector was carried out in order to select the most suitable model for the current task. The following YOLO versions were tested:

- YOLOv5;
- YOLOv6;
- YOLOv8;
- YOLOv9;
- YOLOv10.

For each version, three different model sizes were considered: *nano* (lightest), *medium* (medium size), and *x* (largest). The models are identical in terms of layers but differ in two hyperparameters: network width and depth multipliers. The former determines the number of channels in each layer, and the latter defines the spatial dimensions of the input tensors. All models were trained on the cleaned video set selected in the previous step.

#### 4.4.3. Training Procedure

The models were trained using various data augmentation techniques [32] with the aim of making the models more robust to distortion and preventing overtraining. The following augmentation techniques were used:

- Randomly varying the image saturation.
- Randomly changing the brightness of the image.
- Rotating the image by a random angle.
- Shifting the frame.
- Scaling (zooming in or out) the frame.
- Mirroring a frame (horizontally).
- Combining several frames into one [33].
- Randomly cutting the frame fragments [34].

**Figure 6:**
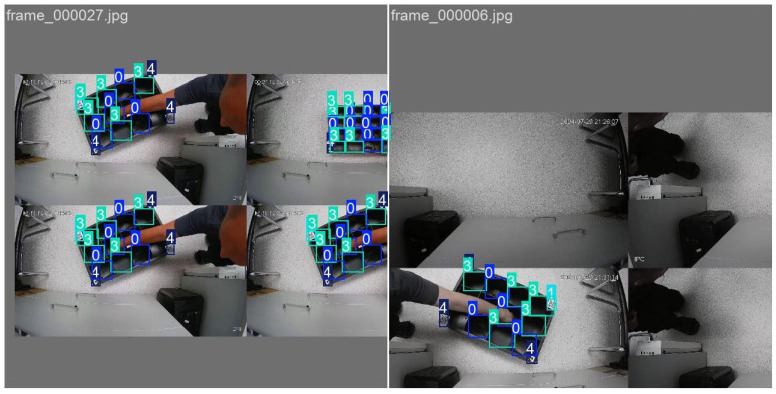
Visualisation of the augmented training examples.

These methods were applied at random, which significantly increased the diversity of the input data. This led to a substantial increase in the number of trained examples, by almost a factor of 100, to 18,462 frames. This, in turn, helped to improve the generalisability of the models. Figure 6 below shows a visualisation of the augmented training examples.

In order to accelerate the training process and enhance its efficiency, we implemented the initialisation of the neural network weights with pre-trained values. In this instance, the weights of the reference network were frozen, with only the final layers of the detector being pre-trained. Labelling smoothing was employed to mitigate marking errors and prevent the adverse impact of incorrect examples, thereby reducing the influence of potential errors in the training data.

The models were trained for 50 epochs. After each epoch, the model weights were stored, and the models that showed the best results according to the ${mAP}_{\left\{50-95\right\}}$ metric were selected for testing.

### 4.5. Training Procedure of the Developed Algorithm

The developed algorithm was tested on the set of images specified in Section 4.4.1, comprising 751 frames, without the application of augmentation.

The VGG network was trained on two classes of cells: empty and part-occupied. The training was performed using an NVIDIA GeForce GTX 1080 Ti graphics (TSMC Samsung, Hsinchu Science Park, Hsinchu, Taiwan) card with 11 GB of video memory, at 40 epochs, with a batch size of four photos. For the training and testing phases, 2853 and 318 fragments were selected from 2940 original images of the training sample, with up to 16 fragments of empty and filled cells in each image.

In this project, separate YOLOv5 neural networks were trained to detect ArUco markers, QR markers, and cells. The training was performed using a Tesla T4 graphics card (TSMC Samsung, Hsinchu Science Park, Hsinchu, Taiwan) with 16 GB of video memory, and 2000 training epochs were completed. For the ArUco detection network, 108 frames were used out of a total number of frames. The QR code detection network was trained using 137 frames out of their total number. The training photos contained two types of containers: plastic with 12 cells (see Figure 2) and wooden with 16 cells. A total of 246 images were used to train the plastic container cell network, and 41 images were used to train the wooden container cell detection network.

## 5. Results

### 5.1. Analysis of Training Results of Single-Channel Moment Detectors

**Figure 7:**
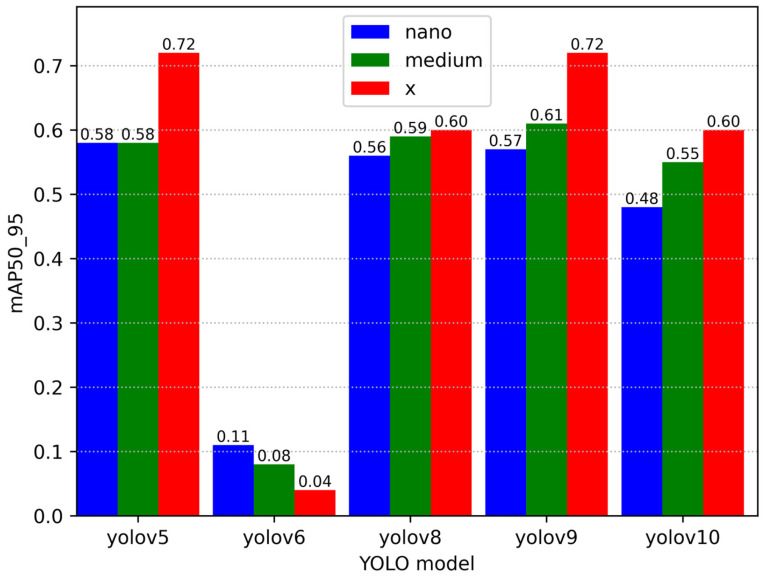
Graph comparing the accuracy of different versions of YOLO models.

Figure 7 provides a visual representation of the accuracy comparison between the different versions and sizes of the YOLO models.

The visualisation in Figure 7 allows you to assess which model performs best at detecting and recognising parts in the shooting conditions in the videos provided.

**Figure 8:**
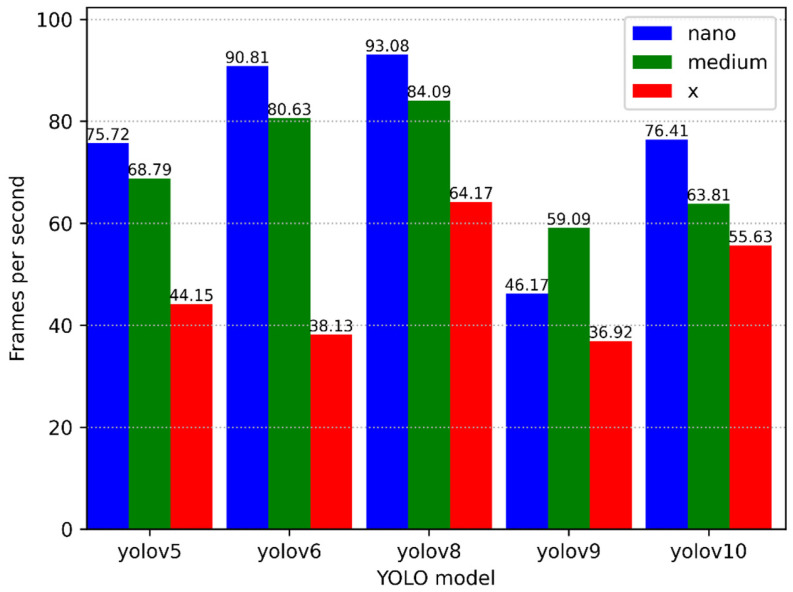
Graph comparing the speed of different versions of YOLO models.

A visualisation of the speed comparison of different versions and sizes of YOLO models for the task of ArUco-marker detection is presented in Figure 8. The comparison was performed on a computer with an Intel Xeon Gold 6258R processor and an RTX3090 video card with 24 GB of video memory.

Following a thorough review of the available research, it has been determined that YOLOv5 and YOLOv9 demonstrate the optimal accuracy-to-speed ratio, making them the preferred choice for the current task. YOLOv6 is not recommended for utilisation due to its suboptimal accuracy despite its high processing speed. YOLOv8 and YOLOv10 exhibit balanced results; however, they are comparatively less favoured in comparison to YOLOv5 and YOLOv9. Consequently, YOLOv5 has been selected for detection purposes.

The trained neural network YOLOv5 was tested on the capabilities of the developed hardware–software complex. The time limit for processing one frame (all classes) was 1.03 s.

### 5.2. Testing the Cascade Algorithm

**Figure 9:**
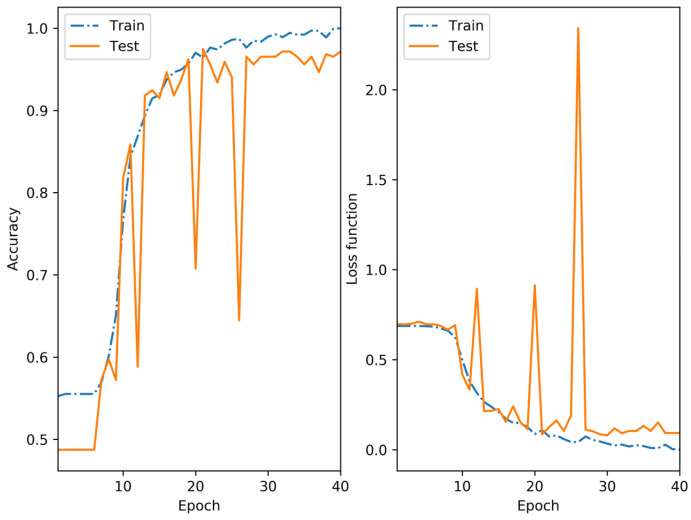
Accuracy metrics and loss functions for training and testing a classification network.

Figure 9 shows the plots of the accuracy ac (8) and the loss function $L_{CE}$ (10) during training and testing of the trained neural network VGG19.

The accuracy of the training sample at epoch 40 was 1.0, with a value of 0.9716 for the test sample. The loss function $L_{CE}$ was 0.0002 and 0.2041 for the training and test samples, respectively.

**Figure 10:**
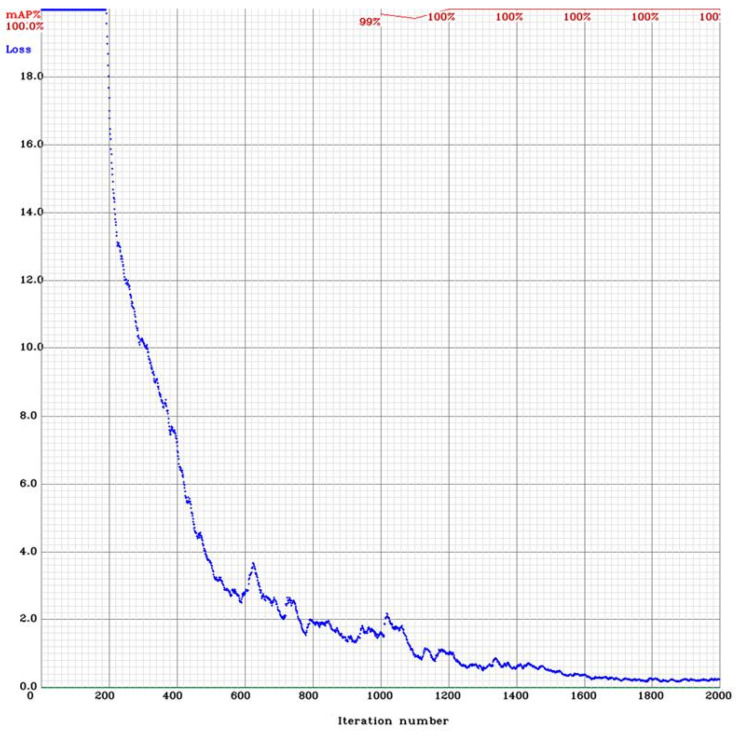
Accuracy and loss function of YOLO training in plastic container cell detection.

Figure 10 shows the graph of the loss function $L_{YOLO}$ (9) and ${mAP}_{\left\{75\right\}}$ (7) when training the plastic container cell detection network.

Table 1 summarises the resulting quality metrics for training YOLO networks.

The study found that the accuracy of the system was consistently high for all objects, with the exception of wooden container cells. However, the accuracy of this cell was higher than that achieved using YOLO on multiple classes. The resulting accuracy of cell detection was complemented by the confidence in the correct classification of the objects in them using the VGG19 neural network. The accuracy results of the developed algorithm (Figure 4) on a test sample of 751 frames are outlined below. The metrics ${mAP}_{\left\{50\right\}}$ (7), ${mAP}_{\left\{75\right\}}$, ${mAP}_{\left\{95\right\}}$, and ${mAP}_{\left\{50-95\right\}}$ (1) for all classes are similar to the results in Section 5.1. The results are summarised in Table 2.

**Figure 11:**
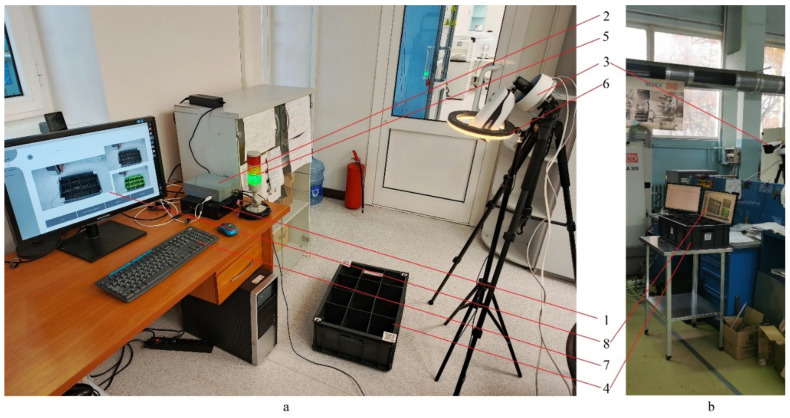
Hardware–software complex in the assembled state in the process of testing: (**a**) in the laboratory room; (**b**) on the production cell. 1—Personal computer; 2—Communication module; 3—IP camera; 4—Container with parts; 5—Signal lamp; 6—LED backlight lamp; 7—Input device; 8—Output device.

Figure 11 shows photos of the developed hardware–software complex in its operation.

**Figure 12:**
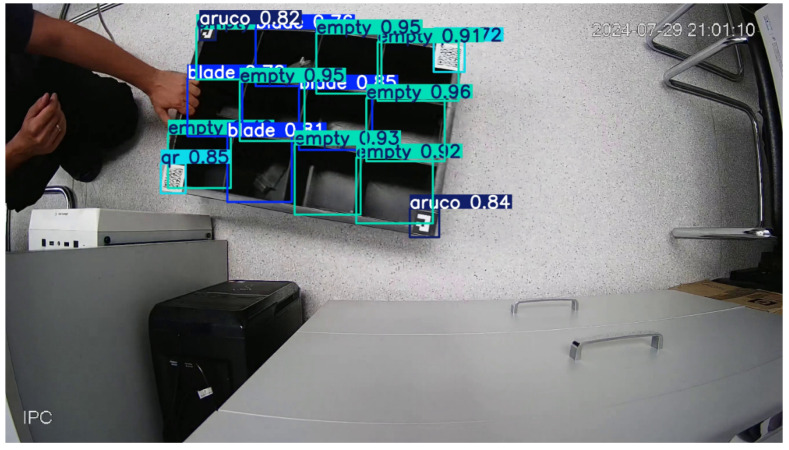
Detection results using the developed complex.

Figure 12 shows one frame with recognised objects obtained during the testing of the hardware–software complex.

The processing time for a single frame using the developed cascade detection algorithm was 3.92 s.

## 6. Discussion

In order to interpret the data obtained as a result of the study, it is necessary to explain once again the similarities and differences between the authors’ approaches and, in general, the vision of the work. Firstly, we will assess the accuracy and speed of the single-channel moment detectors obtained from the experiments as follows:

- YOLOv5: all model sizes (nano, medium, x) demonstrated strong performance, with the x model achieving the highest accuracy of approximately 0.7. This validates the efficacy of this version for the part detection task.
- YOLOv6: it is evident that the accuracy of all model dimensions is significantly lower in comparison to other versions, which consequently makes YOLOv6 a less favoured choice for this task.
- YOLOv8: the YOLOv8 models demonstrate intermediate results, which are inferior to those of the YOLOv5 model but superior to those of the YOLOv6 model. The medium-sized (medium) model has been found to be the most stable.
- YOLOv9: this version demonstrates comparable results to YOLOv5 while exhibiting enhanced stability across diverse model sizes. The accuracy increases from nano to x, with the largest model achieving a result of 0.65.
- YOLOv10: YOLOv10 demonstrates the highest results following YOLOv5, indicating the potential of this version for further utilisation. Notably, model x achieved an accuracy of approximately 0.65.

Comparison of models by speed:

- YOLOv5: the models in this version demonstrate high performance, particularly the nano model, which achieves over 70 frames per second.
- YOLOv6: the YOLOv6 version demonstrates the highest processing speed across all versions, particularly for the nano and medium models, achieving approximately 90 and 80 frames per second, respectively. However, this does not counterbalance the issue of low accuracy.
- YOLOv8: the YOLOv8 version demonstrates comparable results to YOLOv6, exhibiting high-speed performance for nano and medium models.
- YOLOv9: the YOLOv9 model is the slowest of the group, although the largest model is comparable in speed to YOLOv5.
- YOLOv10: demonstrates a balanced processing speed across all sizes.

As stated in the Results section, the YOLOv5 network demonstrated the optimal accuracy-to-speed ratio for detection and was utilised in the developed set.

As shown in Table 3, we can see a comparison of the results for accuracy and speed, along with the training sample size. The accuracy results are shown for a test sample of 751 frames, and these are the same for both approaches. The speed is determined using the computer included in the developed complex.

As a result, a more than twofold reduction in the amount of training data was achieved (not including the amount obtained using the augmentation used in the first approach); up to 300 images were required for each of the neural networks used in the algorithm. At the same time, the accuracy was significantly increased from 0.72 to 0.93, an increase of 29.17%. This demonstrates that the application of the developed cascade algorithm for training convolutional neural networks enhances accuracy while substantially minimising the necessary training sample size. It is also possible to set an inverse problem: with a fixed training sample size, breaking the training process into logical elements, it is possible to perform better training. For example, a container detector can be trained by sampling different light modes and angles to improve its robustness to noise effects. The developed algorithm can be used in various fields where deep learning is employed, such as in computational biology [35], in life sciences [36], and in optimising industrial processes [37].

The use of multiple neural networks significantly reduces the processing speed of incoming information (by almost four times), as it employs three YOLO networks and one VGG network in sequence to classify images within the cells. However, in serial production conditions, as outlined in the Problem section, this is not a major concern. The processing speed is faster than if the information were entered by a human, and full processing is only performed for one or two frames when the container reaches its designated location. Finally, the activation of the complex work occurs at the occurrence of the required event (arrival of containers before processing, collection of containers after processing).

## 7. Conclusions

The developed cascade algorithm, which was used for testing the hardware–software complex, demonstrated a high accuracy of calculation of the state of the production process in the presence of “information noise” and with a relatively small amount of training data. Significant influences on the errors of the proposed algorithm operation are caused by lighting conditions and the violation of the process approach (e.g., the location of the box in the wrong place, its wrong orientation, partial presence in the frame). The first factor is partially eliminated by the automatic switching on of the sub-colour, which is implemented for this purpose in the complex. The second factor is eliminated by implementing lean production principles.

The disadvantage of the proposed solution is that the developed complex in the considered set can work on one workstation.

Further development of the theme is the division of the computer complex into two parts: client (processes only ArUco-markers) and server (performs all other functionality), with further development of stream data processing.

The results of the work are particularly relevant in the conditions of multi-machine service, where the feeding and stacking of workpieces is not fully automated.

## Figures and Tables

**Table 1:** Summary accuracy metrics on training samples.

Detection Object	Precision	Recall	mAP	$L_{YOLO}$
ArUco	1	0.98	1	0.10
QR	0.97	1	1	0.11
Plastic container cells	0.99	0.98	0.99	0.23
Wooden container cells	0.88	0.87	0.87	0.76
ArUco	1	0.98	1	0.10

**Table 2:** Accuracy metrics ${\mathrm{mAP}}_{\left\{t\right\}}$ on the test sample.

Detection Object	${\mathrm{mAP}}_{\left\{50\right\}}$	${mAP}_{\left\{75\right\}}$	${mAP}_{\left\{95\right\}}$	${mAP}_{\left\{50-95\right\}}$
ArUco, QR, parts, empty cells	0.9358	0.9332	0.9319	0.9336

**Table 3:** Comparison of algorithm results and training sample sizes.

Objects of Comparison and Difference	${\mathrm{mAP}}_{\left\{50-95\right\}}$	Frame Processing Speed, s	Total Volume of Training Sample
One YOLO network	0.72	1.03	1906 (18,462 including augmentation)
Developed algorithm	0.93	3.92	836
Absolute difference	0.21	2.89	−1070
Relative difference, %	29.17%	279.65%	−56.14%

**Figure A1:**
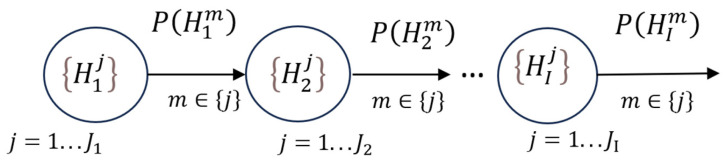
Bayesian network topology for a type 1 network.

**Figure A2:**
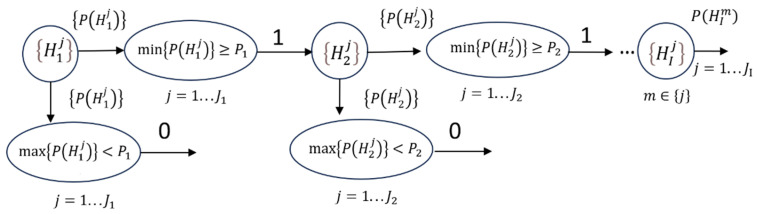
Topology of the second type of network.

## Data Availability

The original data presented in the study are openly available in DataSensors at https://drive.google.com/drive/folders/1o3arlTzPP55WTqchxG67GBwHNjf7Psxj?usp=sharing (accessed on 1 February 2025).

## Appendix A

Suppose that it is necessary to define (detect) $H_{i},\;i=1\ldots I$ events (phenomena or objects), each of which is defined by a sample of $j\ldots J_{i}$ admissible realisations. In essence, we define a set of admissible events $\left\{H_{i}^{j}\right\}=\left\{{\left\{H^{j}\right\}}_{i}\right\},\;i=1\ldots I,\;j=1\ldots J_{i}$ such that for each element $H_{i}^{j}$ of this set, there exists a probability $P\left(H_{i}^{j}\right)$ of its belonging, which is determined by the following relations:

(A1) $$\left(H_{i}^{j}\right)=\left\{P\left(H_{i}^{j}\right),\forall k\;\left|\;k\neq j,j=1\ldots J_{i},\;{\{H}_{i}^{k}\right\}\cap \left\{H_{i}^{j}\right\}=0,P\left(H_{i}^{k}\right)=1-min\{P\left(H_{i}^{j}\right)\}\;\right\},$$

(A2) $$P\left(H_{i+1}^{m}\right)=\left\{P\left(H_{i+1}^{m}\right),\forall i\;|\;i=1\ldots I-1,\;m=1\ldots J_{i+1},\;j=1\ldots J_{i},P\left(H_{i}^{j}\right)=P\left(H_{i}^{j}\right)\;\right\},$$

In other words, at each level *i* there is an admissible set of events $\left\{H_{i}^{j}\right\},\;j=1\ldots J_{i}$, such that the probability of determining any member of the set is equal to $P\left(H_{i}^{j}\right)$. Any event outside this set is inadmissible. The probability of its occurrence is equal to $1-P\left(H_{i}^{j}\right)$. The events of each subsequent level *i* + 1 depend on the previous level *i*. Let us denote the total probability of admissible events of level *i* as $P\left(E_{i}\right)$.

To determine the posterior probability of detecting an admissible event, we will use Bayes’ theorem.

The topology of such a network will have the form shown in Figure A1.

For the initial level *i* = 1, the posterior probability is calculated using the Bayes’ equation:

(A3) $$P\left(H_{i}^{j}/E_{1}\right)=\frac{P\left(H_{i}^{j}\right)\cdot P\left(E_{1}/H_{i}^{j}\right)}{P\left(E_{1}\right)},$$

where

(A4) $$P\left(E_{1}\right)=\sum_{j=1}^{J_{1}}P\left(H_{i}^{j}\right)\cdot P\left(E_{1}/H_{i}^{j}\right),$$

For level i+1, i=1…I−1, the posterior probability is determined by the dependency:

(A5) $$P\left(H_{i+1}^{j}/E_{i+1}\right)=\frac{\sum_{k=1}^{J_{i}}P\left(H_{i+1}^{j}\right)\cdot P\left(H_{i}^{k}/E_{i}\right)\cdot P\left(E_{i+1}/H_{i+1}^{j}\right)}{P\left(E_{i+1}\right)},$$

where

(A6) $$P\left(E_{i+1}\right)=\sum_{j=1}^{J_{i+1}}\sum_{k=1}^{J_{i}}P\left(H_{i+1}^{j}\right)\cdot P\left(H_{i}^{k}/E_{i}\right)\cdot P\left(E_{i+1}/H_{i+1}^{j}\right).$$

Note that the number of probability summands $R_{i+1}$ in the full probability equation $P\left(E_{i+1}\right)$ will be equal according to (A6):

(A7) $$R_{i+1}=J_{1}\cdot J_{2}\ldots \cdot J_{i+1}=\prod_{k=1}^{i+1}J_{k}.$$

Assuming that each additive component of the full probability (A5) corresponds to an element of the training sample for the neural network that detects admissible events at level *I*, the number of elements of this sample must satisfy inequality (A7).

(A8) $$R_{NN}^{1}\geq \prod_{i=1}^{I}J_{i}.$$

We redefine the posterior probability $P\left(H_{i}^{j}/E_{i}\right)$ of the level *i* as follows:

(A9) $$\left\{\begin{array}{c} P\left(H_{i}^{j}/E_{i}\right)=\left\{0,\;\forall i,\forall j|i=1\ldots I,\;j=1\ldots J_{i},\;max\left\{P\left(H_{i}^{j}/E_{i}\right)\right\}<P_{i}\right\},\\ P\left(H_{i}^{j}/E_{i}\right)=\left\{1,\;\forall i,\forall j|i=1\ldots I,\;j=1\ldots J_{i},\;min\left\{P\left(H_{i}^{j}/E_{i}\right)\right\}\geq P_{i}\right\}. \end{array}\right.$$

Thus, the transition from the current level to the next level is complemented by a limit condition. This means that if the maximum posterior probability of detection of any of the events $H_{i}^{j}$ is less than some threshold $P_{i}$, then the transition to the next level is not performed, and on the contrary, if the minimum posterior probability of detection of any of the events $H_{i}^{j}$ is greater than or equal to the threshold $P_{i}$, then the transition is performed.

The topology of such a network is shown in Figure A2.

For such a network, the posterior probabilities at each level *i* are determined by the following relationships:

(A10) $$P\left(H_{i}^{j}/E_{i}\right)=\frac{P\left(H_{i}^{j}\right)\cdot P\left(E_{i}/H_{i}^{j}\right)}{\sum_{j=1}^{J_{i}}P\left(H_{i}^{j}\right)\cdot P\left(E_{i}/H_{i}^{j}\right)},\;i=1\ldots I.$$

The number of detecting neural networks or separately trained fragments of one network required will correspond to the number of training samples for each network or the number of fragments of one global network. The total number of samples will be equal to the number of levels *I*, and the recommended minimum total number of elements of these samples will obey the following dependence:

(A11) $$R_{NN}^{2}\geq \sum_{i=1}^{I}J_{i}.$$

Comparing dependencies (A8) and (A11), we can conclude the advantage of the second type of neural network topology in terms of reducing the training sample size.

(A12) $$K=\frac{R_{NN}^{1}}{R_{NN}^{2}}=\frac{\prod_{i=1}^{I}J_{i}}{\sum_{i=1}^{I}J_{i}}.$$

Let us consider an example of the second type of network, using the detection of the placement of the required part size in container cells as a model. Together with the detection, we will analyse the permissible tare arrangement that is acceptable for solving the problem of detecting the part size. Table A1 summarises the event options.

**Table A1:** Event options for detecting part sizes in the container.

Level, *i*	Name of the Event	$\mathrm{Event}\;\mathrm{Options}\;H_{i}^{j}$
1	Container location	{compliant, non-compliant}
2	Cell perimeter detection	{perimeter defined, perimeter undefined}
3	Filling the container cell	{filled, unfilled}
4	Part size, total *N* permissible sizes	*N*·{permissible size, impermissible size}

The calculation of the index of training sample size reduction for the comparison of networks of the first and second topology types shows that, in accordance with the dependence (A13) for the index *K*, even when one size *N* = 1 is detected, the required sample size will be reduced by half.

(A13) $$K=\frac{R_{NN}^{1}}{R_{NN}^{2}}=\frac{2\cdot 2\cdot 2\cdot 2N}{2+2+2+2N}=\frac{8N}{3+N}.$$
